# Diagnostic Accuracy of a Multi-Target Artificial Intelligence Service for the Simultaneous Assessment of 16 Pathological Features on Chest and Abdominal CT

**DOI:** 10.3390/diagnostics15212778

**Published:** 2025-11-01

**Authors:** Valentin A. Nechaev, Nataliya Y. Kashtanova, Evgenii V. Kopeykin, Umamat M. Magomedova, Maria S. Gribkova, Anton V. Hardin, Marina I. Sekacheva, Varvara D. Sanikovich, Valeria Y. Chernina, Victor A. Gombolevskiy

**Affiliations:** 1 S.S. Yudin City Clinical Hospital, Moscow Department of Health, Moscow 129090, Russia; dfkz2005@gmail.com (V.A.N.);; 2 Multidisciplinary Medical Center of the Bank of Russia, Moscow 117647, Russia; 3 World-Class Reserch Centre “Digital Biodesign and Personalized Healthcare”, I.M. Sechenov First Moscow State Medical University (Sechenov University), Moscow 119991, Russia; 4 Intelligent Radiology Assistance Laboratories LLC (IRA LABS), Moscow 143026, Russia; 5 Northern State Medical University, Arkhangelsk 163000, Russia; 6 AIRI, Moscow 105064, Russia; 7 Russian Medical Academy of Continuous Professional Education, Moscow 125993, Russia

**Keywords:** artificial intelligence, computer vision, computed tomography, chest, abdomen

## Abstract

**Background/Objectives**: Chest, abdominal, and pelvic computed tomography (CT) with intravenous contrast is widely used for tumor staging, treatment planning, and therapy monitoring. The integration of artificial intelligence (AI) services is expected to improve diagnostic accuracy across multiple anatomical regions simultaneously. We aimed to evaluate the diagnostic accuracy of a multi-target AI service in detecting 16 pathological features on chest and abdominal CT images. **Methods**: We conducted a retrospective study using anonymized CT data from an open dataset. A total of 229 CT scans were independently interpreted by four radiologists with more than 5 years of experience and analyzed by the AI service. Sixteen pathological features were assessed. AI errors were classified as minor, intermediate, or clinically significant. Diagnostic accuracy was evaluated using the area under the receiver operating characteristic curve (AUC). **Results**: Across 229 CT scans, the AI service made 423 errors (11.5% of all evaluated features, *n* = 3664). False positives accounted for 262 cases (61.9%) and false negatives for 161 (38.1%). Most errors were minor (62.9%) or intermediate (31.7%), while clinically significant errors comprised only 5.4%. The overall AUC of the AI service was 0.88 (95% CI: 0.87–0.89), compared with 0.78–0.81 for radiologists. For clinically significant findings, the AI AUC was 0.90 (95% CI: 0.71–1.00). Diagnostic accuracy was unsatisfactory only for urolithiasis. **Conclusions**: The multi-target AI service demonstrated high diagnostic accuracy for chest and abdominal CT interpretation, with most errors being clinically negligible; performance was limited for urolithiasis.

## 1. Introduction

Artificial intelligence (AI) technologies have introduced novel opportunities in radiology, including enhanced diagnostic accuracy, workflow optimization, and the advancement of medical research [1,2]. The integration of AI algorithms into clinical practice is necessitated by the steadily increasing volume of imaging studies, the persistent shortage of radiologists, and the ongoing demand for greater diagnostic precision in imaging modalities [1,2]. Among these modalities, contrast-enhanced computed tomography (CT) of the chest, abdomen, and pelvis remains one of the most accessible and accurate techniques. CT is recommended for the assessment of tumor extent, treatment planning, and evaluation of therapeutic efficacy and is therefore considered an indispensable component of comprehensive patient management [3]. Nonetheless, radiology reports based on chest CT [4] and abdominal CT [5] are frequently subject to diagnostic errors, most commonly false-negative findings. In this context, the implementation of multipurpose AI-based systems capable of detecting pathological changes across multiple anatomical regions concurrently appears highly relevant. Such systems not only have the potential to reduce diagnostic error rates but may also contribute to mitigating the increasing clinical workload of radiologists [6]. However, the diagnostic performance of AI tools requires rigorous evaluation, including systematic assessment of potential sources of error and identification of limitations that may constrain their widespread clinical adoption [2].

## 2. Materials and Methods

### 2.1. The Study Design

**Figure 1:**
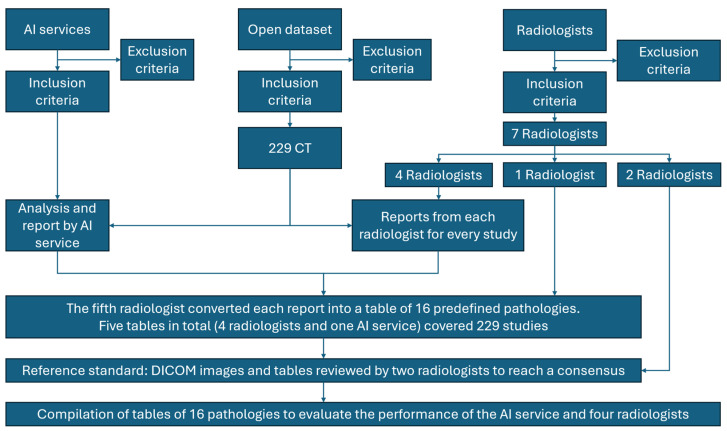
Study Design. From 250 CT examinations screened, 21 were excluded for technical/protocol deviations, yielding 229 studies. Four radiologists independently interpreted each study; one compiler radiologist converted all reports (4 radiologists + AI) into standardized tables; two expert referees reviewed original DICOM images and tables to establish the consensus reference standard. Performance of AI and radiologists was then evaluated against this reference.

This single-center retrospective diagnostic accuracy study followed the CLAIM and STARD guidelines [7,8]. The study workflow is illustrated in Figure 1. Prior to the analysis, dataset preparation and radiologist training were performed according to predefined inclusion and exclusion criteria.

### 2.2. Study Registration

This retrospective diagnostic accuracy study analyzed publicly available, anonymized CT data and therefore did not require separate protocol registration (e.g., ClinicalTrials.gov). Ethical approval was obtained from the local ethics committee of Moscow City Clinical Hospital No. 1. (protocol dated 1 March 2024). The BIMCV-COVID-19+ dataset had prior approval from the Hospital Arnau de Vilanova ethics committee (CElm 12/2020, Valencia, Spain) and was funded through regional and EU Horizon 2020 grants. All data were anonymized before release, so informed consent was waived.

### 2.3. Data Source

Anonymized CT scans were acquired in 2020. Retrospective evaluation by radiologists and the AI system was carried out between 14 March 2024, and 2 November 2024. All CT examinations originated from the publicly available BIMCV-COVID-19+ dataset (Valencia Region, Spain), collected in 2020 from 11 public hospitals and standardized to UMLS terminology [9].

#### 2.3.1. Inclusion Criteria

Adult chest and abdominal CT images; slice thickness ≤ 1 mm; scan coverage extending from the lung apices to the ischial bones, acquired during deep inspiratory breath-hold.

#### 2.3.2. Exclusion Criteria

CT studies with protocol deviations (slice thickness > 1 mm or incomplete anatomic coverage), severe motion or beam-hardening artifacts, non-standard patient positioning, or upload/parse failures. Studies with corrupted DICOM tags or missing key series were also excluded.

### 2.4. Data Preprocessing

No additional data preprocessing was applied beyond standard DICOM parsing of the publicly available BIMCV-COVID-19+ dataset; images were analyzed as provided.

### 2.5. Data Partitions

#### 2.5.1. Assignment of Data to Partitions

All 229 eligible CT examinations were used solely as an independent test set for the locked AI system. No additional training or validation split was performed.

#### 2.5.2. Level of Disjointness Between Partitions

Each examination (one patient) was treated as a single unit of analysis; no overlap existed between cases.

### 2.6. Intended Sample Size

The required sample size was calculated a priori as 236 examinations to estimate sensitivity and specificity with 95% confidence and ±10% error. To account for possible data loss (artifacts, upload failures, or absent non-contrast series), 250 CTs were selected; after exclusions, 229 remained for final analysis.

### 2.7. De-Identification Methods

All CT examinations were provided as part of the publicly available BIMCV-COVID-19+ dataset, which had been fully anonymized by the data provider before release. Personal identifiers—including patient name, date of birth, medical record numbers, and examination dates—were removed from DICOM headers. Only anonymous study IDs linking imaging data to accompanying radiology reports were retained for analysis. The de-identification procedure of the BIMCV dataset was reviewed and approved by the local ethics committee (CElm: 12/2020, Valencia, Spain).

### 2.8. Handling of Missing Data

Studies lacking mandatory series (e.g., contrast-enhanced scans without a corresponding non-contrast series) or with missing annotations/upload failures were treated as technical exclusions. No additional data imputation or image reconstruction was performed; such cases were not included in endpoint analyses.

### 2.9. Image Acquisition Protocol

Detailed scanning parameters can be obtained from the original publicly available dataset [9]. Examinations were performed on multislice CT scanners routinely used in hospitals of the Valencia region. Slice thickness was ≤1 mm, and coverage extended from the lung apices to the ischial bones. Scans were obtained during deep-inspiration breath-hold. Both non-contrast and contrast-enhanced studies were included; contrast-enhanced examinations lacking a non-contrast series were excluded. Detailed scanner models, reconstruction kernels, and exposure parameters were not available in the public dataset; all scans followed standard clinical protocols for chest–abdomen–pelvis CT in the region.

### 2.10. Human Readers

Seven radiologists participated in the study. Inclusion criteria comprised at least three years of experience in interpreting chest and abdominal CT scans. Exclusion criteria included failure to complete calibration or training according to the study protocol. The radiologists were assigned to three roles: annotators (*n* = 4), compiler (*n* = 1), and referees (*n* = 2). The annotators, all board-certified radiologists with 5–8 years of experience, independently interpreted all CT examinations using RadiAnt DICOM Viewer 2023.1 outside their routine clinical duties. Prior to the study, all readers attended a short calibration session with sample clinical cases to standardize interpretation criteria. Case order was randomized individually, and readers were blinded to AI outputs, clinical data, and one another’s results. For each of the 16 predefined pathologies, findings were recorded for ROC AUC analysis. The compiler (8 years of experience) reviewed all radiologist and AI reports to compile structured tables containing the same 16 pathologies, resulting in five tables (four radiologists and one AI system) covering 229 CT studies. The referees (each with over 8 years of experience) independently reviewed DICOM images and resolved discrepancies by consensus, after which they were granted access to all five result tables for comparative evaluation.

### 2.11. Annotation Workflow

Each of the six radiologists (annotators, *n* = 4; referees, *n* = 2) reviewed every CT slice of all 229 examinations to ensure comprehensive assessment. The compiler (*n* = 1) subsequently analyzed the outputs from the four annotators and from the AI system (IRA LABS AI service), which processed the same 229 CT studies and produced both DICOM SEG annotations and DICOM SR structured reports. For each study, the compiler registered the presence or absence (binary classification) of all 16 predefined pathologies.

### 2.12. Reference Standard

The reference standard for performance evaluation was the consensus of two senior radiologists (>8 years’ experience) who were not involved in the initial readings. They independently reviewed all CT examinations without access to AI outputs or initial reader reports using RadiAnt DICOM Viewer 2023.1. Disagreements were resolved by consensus or, if unresolved, by a third adjudicator. Formal inter-reader variability was not a primary objective; however, prior work highlights substantial variability in CT reporting [10].

Expert annotations for all 229 examinations were documented in a standardized table covering 16 predefined pathological features. Performance comparisons were made among: (1) initial annotations from four radiologists, (2) AI system outputs, and (3) the expert consensus reference standard.

For exploratory error analysis only, the same experts re-examined cases after reviewing AI outputs to categorize AI detections and errors; these post-AI reviews were not used to generate reference standard metrics.

### 2.13. Model

#### 2.13.1. Model Description

A multipurpose AI service (IRA LABS, registered medical device RU №2024/22895) was used for simultaneous detection of 16 predefined pathologies on chest–abdominal CT (Table 1).

The final release version (v6.1, January 2024) identical to the one deployed in clinical practice was applied without retraining or parameter changes. Input consisted of DICOM CT series; output was generated as DICOM SEG annotations and DICOM SR structured reports.

#### 2.13.2. AI Service Inclusion and Exclusion Criteria

##### Inclusion Criteria

Software registered as a certified medical device (MD) utilizing artificial intelligence (AI) technology in the official national registry of medical software. Software tested and validated within the Moscow Experiment—a large-scale governmental initiative for clinical deployment of computer vision AI in radiology [13]. Demonstrated diagnostic performance with ROC AUC ≥ 0.81 for each target pathology, in accordance with methodological recommendations [11,12]. Capability to analyze both chest and abdominal CT examinations within a single inference pipeline.

##### Exclusion Criteria

AI products whose participation in the Moscow Experiment was suspended, discontinued, or failed official performance verification [13]. Systems limited to single-region analysis (e.g., chest-only AI) or lacking multiclass pathology detection capability.

The IRA LABS AI service (version 6.1, January 2024) was selected because it fulfilled all inclusion criteria and provided the broadest pathology coverage among eligible AI services participating in the Moscow Experiment [13].

#### 2.13.3. Software and Environment

The proprietary system was executed as an off-the-shelf product. Internal architecture, model parameters, and potential ensemble methods are not publicly disclosed by the developer. Inference was run on a workstation with AMD Ryzen 7 7700, 64 GB RAM, 480 GB SSD, and NVIDIA RTX 4060 (8 GB) GPU, using Ubuntu 22.04.

#### 2.13.4. Initialization of Model Parameters

The pre-trained production version of the AI model was used as released by the developer. No fine-tuning, weight reinitialization, or hyperparameter modification was performed for this study. The operating point (decision thresholds) was the vendor’s default, locked a priori and not tuned on the test set.

#### 2.13.5. Training

##### Details of Training Approach

No additional training or fine-tuning was performed for this study. The AI service was applied as an off-the-shelf production version (v6.1, January 2024) identical to the clinically deployed release.

##### Method of Selecting the Final Model

The version used was previously chosen and locked by IRA LABS during its clinical validation within the Moscow Experiment on computer-vision technologies. No modifications were made for the present evaluation [13].

##### Ensembling Techniques

The developer has not disclosed whether internal ensemble methods were used. For this study, a single instance of the AI service was applied for CT analysis, providing DICOM SEG annotations and structured text reports.

### 2.14. Evaluation

#### 2.14.1. Metrics

Diagnostic performance of the AI system and radiologists was quantified using the area under the ROC curve (AUC, 95% CI via DeLong) [14]. For each of the 16 pathologies, True positives (TP), false positives (FP), true negatives (TN), and false negatives (FN) counts were recorded, and classification errors stratified as minor, intermediate, or major.

#### 2.14.2. Robustness Analysis

AUC was additionally calculated for clinically significant findings only to assess robustness for critical pathologies.

#### 2.14.3. Methods for Explainability

AI outputs were interpreted via DICOM SEG visualizations and structured text reports. Errors were cross-checked against the reference standard and categorized by clinical significance.

#### 2.14.4. Data Independence

BIMCV-COVID-19+ had not been used in model training; all 229 CT studies were independent of the AI development data. Testing was limited to this single external dataset; no further external validation was available.

#### 2.14.5. Comparison and Evaluation Methodology

Comparative analysis was performed using five structured 229 × 16 matrices (four human readers and one AI system), each containing binary presence/absence determinations for all predefined pathologies. These matrices were compared with the expert consensus reference standard established by the two referees to derive the metrics described above.

For pathologies with quantitative thresholds (Table 1), the AI system applied predefined anatomical cut-offs (e.g., ≥40 mm for ascending aorta) based on DICOM SR measurements, while radiologists relied on visual assessment and manual measurements.

#### 2.14.6. Conversion of AI Outputs to Binary Labels

The fifth radiologist (compiler) converted the AI outputs into binary presence/absence labels for all 16 pathologies. Binary classification was based primarily on DICOM SR measurements and text findings, cross-referenced against thresholds specified in Table 1. DICOM SEG masks served as visual confirmation but were not used for independent volumetric analysis.

Example 1: Aortic dilatation/aneurysm The DICOM SR contained a quantitative measurement: “Ascending aorta diameter: 42 mm.” According to the predefined threshold (≥40 mm for ascending aorta, Table 1), this measurement was classified as “positive” for aortic dilatation. Cases with measurements below threshold were labeled “negative.”

Example 2: Pulmonary nodules The DICOM SEG file displayed a 3D segmentation mask outlining a pulmonary nodule. The accompanying DICOM SR reported: “In the right lung, a 10 × 8 mm node (average size 9 mm according to Fleischner).” Since the threshold is ≥6 mm (Table 1), this case was classified as “positive.” The compiler visually verified that the segmentation mask adequately encompassed the nodule before assigning the label.

#### 2.14.7. Handling of Small Segmentations and Borderline Cases

Segmentations that partially extended beyond lesion boundaries were accepted if the DICOM SR measurement remained above the predefined threshold (Table 1) and the core pathology was adequately captured. Cases where segmentation contours substantially misrepresented the pathology (e.g., including adjacent structures or anatomically incorrect regions) were flagged as false positives during expert consensus review. No additional voxel-based or volumetric filtering beyond the diameter thresholds specified in Table 1 was applied.

### 2.15. Outcomes

#### 2.15.1. Primary Outcome

TP, FP, TN, and FN for AI and human readers in detecting each of the 16 predefined pathologies (Table 1). All errors were stratified by clinical significance:

- Minor—no change in patient management or follow-up needed (examples: missed simple cysts < 5 mm, false-positive osteosclerosis misclassified as rib fracture).
- Intermediate—unlikely to affect primary disease treatment but requiring further testing or follow-up (examples: false-positive enlarged lymph nodes, over-detection of small pulmonary nodules).
- Major—likely to change treatment strategy or primary diagnosis (examples: missed liver/renal masses, missed intrathoracic lymphadenopathy suggestive of metastases).

Expert radiologists assigned these classifications during consensus review based on potential impact on clinical decision-making.

#### 2.15.2. Secondary Outcomes

Area under the ROC curve (AUC) with 95% confidence intervals for each pathology and for the aggregated set, for both AI and radiologists.

Comparative analysis of AI versus radiologists using multi-reader multi-case (MRMC) methods.

Exploratory error review by experts after viewing AI outputs to categorize AI detections (not used as reference standard).

### 2.16. Sample Size Calculation

The minimum required sample size was estimated using the formula for a single proportion: *n* = Z^2^ × P × (1 – P)/d^2^, where Z = 1.96 (95% confidence), P = 0.81 (expected sensitivity/specificity), and d = 0.10 (margin of error). This yielded approximately 59 cases with pathology. Assuming an average disease prevalence of 25% across the evaluated pathologies, the total required sample size was *N* = 59/0.25 ≈ 236 examinations. To compensate for potential data loss (artifacts, annotation errors) and the multifocal nature of the study (chest + abdomen), the planned sample was increased to 250. After exclusions, 229 studies remained for final analysis.

### 2.17. Statistical Analysis

Statistical analysis was performed using RStudio (version 2025.09.2+418; Posit Software, PBC, Boston, MA, USA) [15] with the *irr* [16] and *pROC* [17] packages. Data visualization was carried out with GraphPad Prism version 10.2.2 (GraphPad Software Inc., San Diego, CA, USA) [18]. Descriptive statistics were reported as absolute numbers (*n*) and proportions (%). Diagnostic performance was assessed using ROC analysis with calculation of AUC and 95% confidence intervals via DeLong’s method [14]. Comparisons between AI and radiologists used multi-reader multi-case DBM/OR analysis (*RJafroc*) [19]. To control for multiple testing across 16 pathologies, Benjamini–Hochberg correction (q = 0.05) was applied. A two-sided *p* < 0.05 was considered statistically significant.

## 3. Results

### 3.1. Overall Diagnostic Performance

In 229 chest–abdominal CT examinations independently interpreted by four radiologists and the AI system, the AI system produced 423 errors (11.5% of all evaluated features). The prevalence of evaluated pathologies ranged from 9.6% (urolithiasis) to 70.7% (coronary artery calcium) (Table 2).

**Figure 2:**
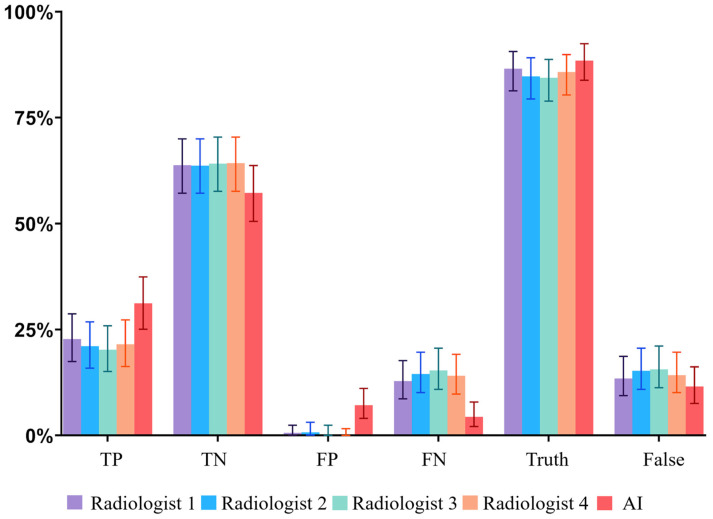
Bar charts showing the relative numbers of TP, TN, FP, FN, true (TP + TN) and false (FP + FN) responses; whiskers = 95% CI. AI exhibited a more liberal operating point (higher FP, lower FN) than individual radiologists.

False positives predominated (*n* = 262; 61.9%) over false negatives (*n* = 161; 38.1%), whereas radiologists showed more false negatives (470–562) than false positives (5–27) (Figure 2). Contingency tables (TP, FP, TN, FN) for each pathology across all features, 4 radiologists, and the AI are provided in Supplementary Files (Tables S1 and S2).

### 3.2. Clinical Significance of Errors

**Figure 3:**
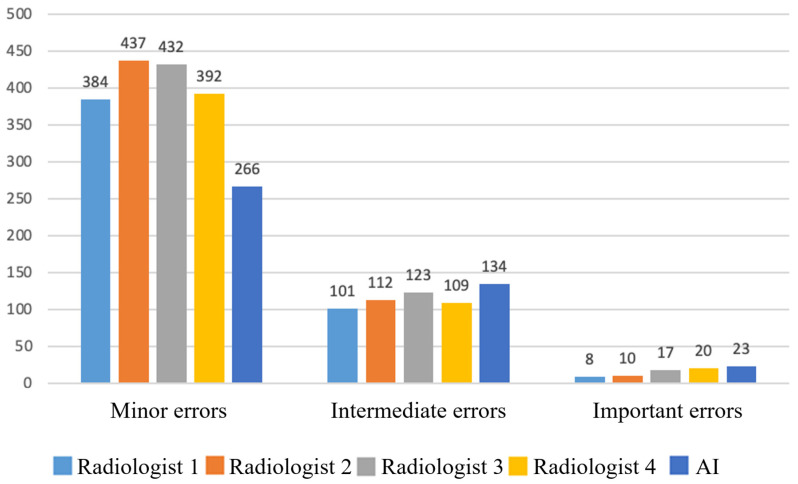
Absolute numbers of false responses (FP + FN) by clinical significance for physicians and AI. Only 5.4% of AI errors were classified as important; ≈0.63% of all assessed instances represented clinically important AI errors.

All errors were stratified by clinical significance. For both radiologists and AI, minor errors were most frequent, followed by intermediate, with major errors being rare (Figure 3).

### 3.3. Breakdown of Clinically Significant AI Errors

**Figure 4:**
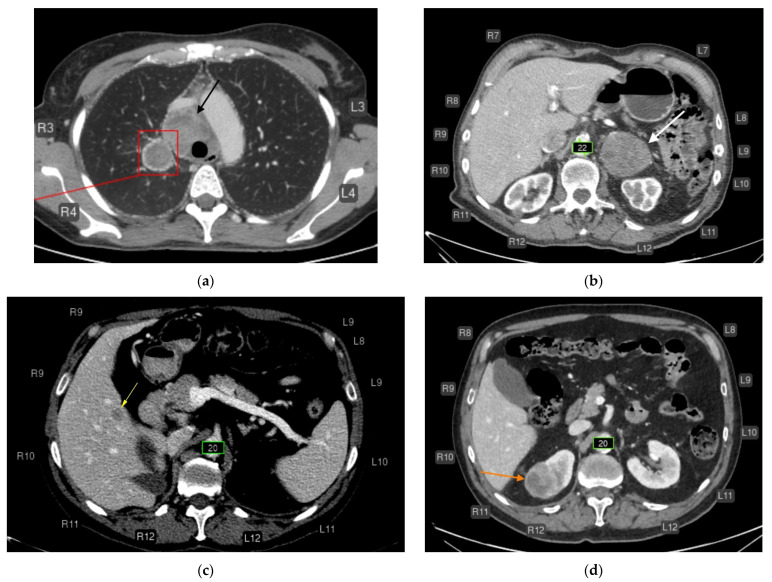
CT of the chest (**a**) and abdomen (**b**–**d**) after AI processing. Clinically significant findings missed by the AI that could substantially affect patient outcomes (false-negative errors). No segmentation (missed pathology): (**a**) Enlarged intrathoracic lymph nodes (black arrow), possibly representing metastases. False-negative error by the AI service. In addition, the AI correctly labeled the pulmonary lesion and rib numbering. (**b**) Left adrenal mass (white arrow), possibly a tumor or metastasis. False-negative error by the AI service. The AI correctly measured the abdominal aortic diameter and labeled rib numbering. (**c**) Hypovascular liver mass (yellow arrow), possibly a metastasis. False-negative error by the AI service. The AI correctly measured the abdominal aortic diameter and labeled rib numbering. (**d**) Right renal mass (orange arrow), possibly a tumor. False-negative error by the AI service. The AI correctly measured the abdominal aortic diameter and labeled rib numbering.

Major errors (false negatives, *n* = 20):

- Liver lesions (8);
- Renal lesions (2);
- Adrenal lesions (2);
- Impaired lung aeration (atelectasis, 2);
- Enlarged intrathoracic lymph nodes (3);
- Pulmonary nodule (1);
- Low vertebral body density (1);
- Urolithiasis (1) (Figure 4).

**Figure 5:**
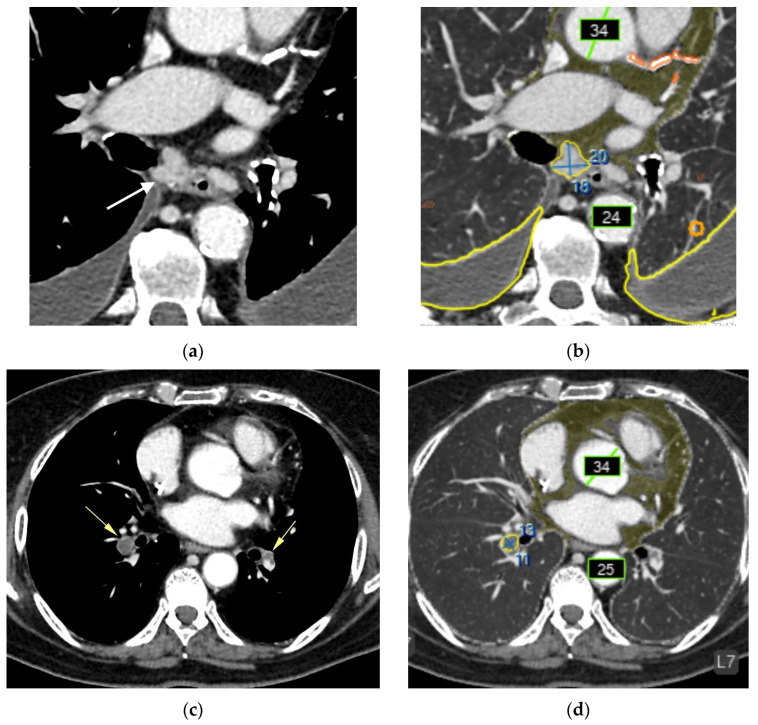
Chest CT before (**a**,**c**) and after AI processing (**b**,**d**). Intermediate errors. (**a**,**c**) Two adjacent, non-enlarged bifurcation lymph nodes (white arrow) are segmented by the AI as a single enlarged node (false-positive error). Although a radiologist can recognize this error, it may be challenging for less experienced readers. The AI service correctly identified bilateral pleural effusion (blue contour), measured the diameters of the ascending and descending thoracic aorta (green), coronary calcifications (orange), paracardial fat (dark green), emphysema (brown), and a small focus of pulmonary infiltration associated with COVID-19. (**b**,**d**) A thrombus in the right pulmonary artery (yellow arrow) is misclassified as enlarged intrathoracic lymph nodes (false-positive error). The AI was not trained to detect pulmonary artery thrombi but was trained to identify enlarged lymph nodes in this region. Due to limited training examples containing both lymphadenopathy and thrombi, the model misinterpreted the lesion as lymph node enlargement. Nevertheless, the detected abnormality could prompt a radiologist to recognize a potential thrombus. The AI correctly measured the diameters of the ascending and descending thoracic aorta (green), coronary calcifications (orange), and paracardial fat (dark green).

Intermediate errors (mostly false positives, *n* = 91):

- Intrathoracic lymph nodes (16);
- Pulmonary nodules (15);
- Impaired aeration (15);
- Aortic dilatation/aneurysm (10);
- Adrenal thickening (10) (Figure 5).

**Figure 6:**
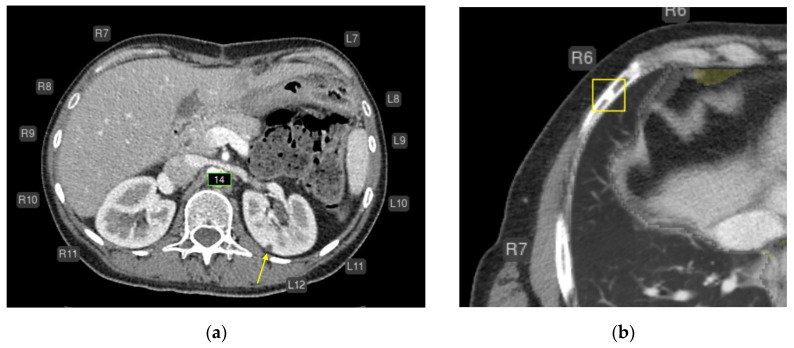
Axial CT of the abdomen (**a**) and chest (**b**) after AI processing. Missed findings without clinical significance. (**a**) A simple cyst in the left kidney measuring approximately 4–5 mm (yellow arrow) was not segmented (false-negative error). This is most likely a simple cyst that does not require follow-up. The AI correctly measured the abdominal aortic diameter (green) and labeled rib numbering. (**b**) A focal area of osteosclerosis in the anterior segment of the right 6th rib was highlighted as a consolidated fracture (yellow box; false-positive error). This finding does not require follow-up. The AI correctly labeled rib numbering and measured the aortic diameter.

Minor errors (*n* = 266):

- Missed small simple cysts (<5 mm) in kidney or liver (70 cases) (Figure 6a);
- Incorrect segmentation of rib fractures (32 cases) misclassified from bone islands, artifacts, or costal cartilage transitions (Figure 6b).

### 3.4. ROC Analysis and AUC Values

AUCs were calculated for each pathology and in aggregate for radiologists and AI (Table 3).

**Figure 7:**
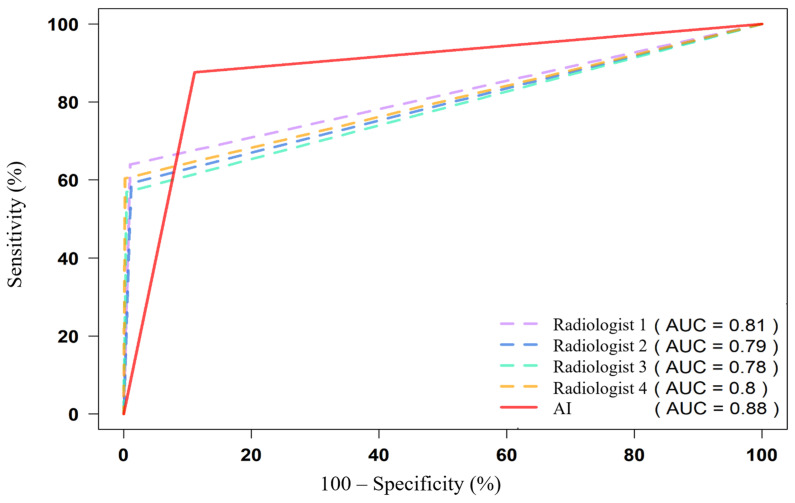
ROC curves comparing radiologists and AI for all features. The AI shows excellent discrimination for vascular, osseous, and emphysema targets, with lower performance on urolithiasis and small solid-organ lesions.

Aggregate AUC: AI = 0.88; highest radiologist = 0.81 (Figure 7).

Clinically significant findings only: mean AI AUC = 0.90 (Table 4).

### 3.5. Diagnostic Performance Categories

According to standard diagnostic categories, AI performance was predominantly excellent or good.

Urolithiasis was the only feature where AI showed inadequate performance.

Radiologists demonstrated poor or inadequate performance for several features, including:

- Aortic dilatation/aneurysm;
- Vertebral compression fractures;
- Rib fractures;
- Pulmonary artery dilatation;
- Low vertebral body density;
- Increased epicardial fat volume here.

## 4. Discussion

In this multi-reader evaluation of 229 chest and abdominal CT examinations comprising 3664 feature-level assessments, the multi-target AI service achieved an aggregate AUC of 0.88 (95% CI 0.87–0.89), outperforming the four independent radiologists (AUC 0.78–0.81) (Table 2, Figure 7). Diagnostic performance was good to excellent for most of the 16 predefined targets. The AI demonstrated clear advantages in vascular, osseous, and morphometric findings, while showing relative deficits for solid-organ masses and airspace disease. The only unsatisfactory target was urolithiasis (AUC ≈ 0.52), which persisted in sensitivity analysis (AUC 0.55). Across modalities, commercial AI shows target-dependent performance, e.g., for airspace disease on chest radiographs [20].

Although the AI system produced more false positives than false negatives (61.9% vs. 38.1%), clinically important AI errors were rare—only 0.63% of all assessed instances—and were mainly missed focal lesions. Stratification of errors revealed that 94.6% of AI mistakes were minor or intermediate. Intermediate false positives often reflected adjacency or merging artifacts and vascular–nodal confusion, whereas minor errors were primarily tiny cysts and rib fracture overcalls. The AI’s superiority in measuring diameters, densities, calcifications, and vertebral deformities likely stems from stable morphometric cues, whereas parenchymal textures and small solid-organ lesions remain more challenging. Its underperformance in urolithiasis plausibly relates to protocol sensitivity: many cases were contrast-enhanced only, whereas optimal stone detection requires non-contrast CT [21].

Although our final sample size (*n* = 229) was slightly below the a priori calculated requirement (*n* = 236), the impact on precision was minimal, increasing the margin of error from ±10.0% to ±10.2%. The narrow confidence intervals observed for most AUC estimates suggest adequate statistical power was maintained.

Our aggregate results align with recent meta-analyses [22,23] reporting that state-of-the-art imaging AI reaches AUC ≈ 0.86–0.94 and, in selected tasks, can match or surpass individual radiologists [23,24]. The complementary patterns of liberal AI (more FP) and conservative readers (more FN) suggest that hybrid strategies—such as triage or double-reading—may reduce important misses while managing FP burden [24]. Incorrect AI outputs can influence readers’ decisions, underscoring the need for guardrails in hybrid workflows [25]. Similar FP–FN patterns have been synthesized in systematic reviews of AI error characteristics [26].

### 4.1. Strengths and Limitations

Key strengths include:

Multi-reader design with blinded interpretation and expert consensus reference standard;

Simultaneous evaluation of 16 diverse targets, enabling a comprehensive view of performance;

Stratification of errors by clinical significance, which provides insights beyond raw sensitivity and specificity;

Interpretive pitfalls and potential automation bias necessitate procedural safeguards and reader training [27].

Limitations include:

Single external dataset, limiting generalizability across protocols and institutions;

Protocol heterogeneity within BIMCV-COVID-19+ and incomplete non-contrast phases for some studies;

Lack of transparency regarding the proprietary model’s architecture and potential ensembling strategies;

The perfect ROC AUC scores (1.00) reflect the small sample size of clinically significant cases available for analysis, rather than necessarily indicating superior model performance. These findings require validation with substantially larger datasets.

Translation from curated datasets to clinical applicability can vary across modalities and tasks [28];

Absence of formal inter-reader variability analysis and external validation on independent cohorts. Limitations of AI services observed in radiography evaluations further argue for protocol-aware validation [29].

Binary classification prioritized clinical relevance over volumetric precision. No quantitative segmentation assessment (e.g., Dice coefficient) was performed; minor imperfections were tolerated if DICOM SR measurements exceeded thresholds. This pragmatic approach may introduce measurement variability and warrants future voxel-level validation.

### 4.2. Public Health Implications

Use of multi-target CT AI may improve early detection of significant findings, speed up reporting, and optimize resource use—critical for systems facing radiologist shortages. Such deployment could enhance population-level outcomes and reduce costs from delayed diagnoses, but requires careful monitoring of error profiles, protocol harmonization, and adherence to evidence-based standards to ensure equitable, safe benefits.

### 4.3. Future Directions

Perform multi-center replication with protocol-aware validation to ensure robustness across scanners and acquisition techniques;

Explore operating point calibration or task-specific thresholds to optimize the FP–FN balance;

Develop targeted refinements for small solid-organ lesions, parenchymal textures, and urolithiasis detection;

Investigate workflow integration strategies, including triage, double reading, or AI-assisted decision support, to translate performance gains into clinical benefit.

Evaluate the economic and public health impact of multi-target AI deployment, including cost-effectiveness analyses, resource allocation, and potential reductions in population-level morbidity and healthcare expenditures.

## 5. Conclusions

A clinically deployed multi-target AI service demonstrated high diagnostic accuracy on chest and abdominal CT across 16 predefined features, outperforming individual radiologists on several vascular, osseous, and morphometric targets while underperforming on urolithiasis and small solid-organ lesions. Clinically important AI errors were rare and predominantly involved missed focal lesions. These findings support the use of multi-target AI as a complementary second reader, provided protocol alignment is ensured and error profiles are prospectively monitored. Future multi-center validations and workflow studies are warranted to confirm generalizability and define optimal integration strategies in routine practice.

## Figures and Tables

**Table 1:** Thresholds for pathologic changes assessed by the multi-target AI service. Thresholds were defined according to institutional methodological guidance [11,12].

Abnormalities	Thresholds
Pulmonary nodules	Presence of at least one pulmonary nodule or lesion larger than 6 mm in short-axis diameter
Airspace opacities (including consolidations/infiltrates)	Presence of any size/volume
Emphysema	Presence of any volume
Aortic dilatation/aneurysm	For aortic dilatation, the threshold diameters were defined as follows: ≥40 mm for the ascending aorta and aortic arch, ≥30 mm for the descending thoracic aorta, ≥25 mm for the abdominal aorta. For aortic aneurysm, the threshold was defined as a diameter > 55 mm in any segment.
Pulmonary artery dilatation	>30 mm in diameter
Coronary artery calcium	Agatston score > 1
Enlarged intrathoracic lymph nodes	Presence of at least one intrathoracic lymph node enlarged to >15 mm in short-axis diameter
Adrenal thickening	Presence of thickening >10 mm
Urolithiasis	Presence of at least one urinary calculus
Rib fractures	Presence of at least one fracture
Low vertebral body density	Attenuation < +150 HU
Vertebral compression fractures	Vertebral body deformity > 25% (Genant grade 2)

**Table 2:** Prevalence of Pathological Features in the Study Dataset (*N* = 229).

Pathology	Positive Cases (*n*)	Negative Cases (*n*)	Prevalence (%)
Coronary artery calcium	162	67	70.7
Renal lesions	148	81	64.6
Airspace opacities	144	85	62.9
Low vertebral density	126	103	55.0
Emphysema	98	131	42.8
Liver lesions	97	132	42.4
Pulmonary nodules	96	133	41.9
Pleural effusion	73	156	31.9
Adrenal thickening	69	160	30.1
Rib fractures	63	166	27.5
Enlarged intrathoracic lymph nodes	55	174	24.0
Pulmonary artery dilatation	54	175	23.6
Vertebral compression fractures	51	178	22.3
Epicardial fat	38	191	16.6
Aortic dilatation/aneurysm	32	197	14.0
Urolithiasis	22	207	9.6

**Table 3:** Area under the ROC curve for diagnostic performance of four radiologists and AI.

Pathology (Number of True Positive Findings from 229)	AUC [95% CI]				
	Radiologist 1	Radiologist 2	Radiologist 3	Radiologist 4	AI
Enlarged intrathoracic lymph nodes (55)	0.952 [0.916–0.989]	0.964 [0.932–0.996]	0.891 [0.836–0.946]	0.936 [0.892–0.981]	0.854 [0.803–0.904]
Aortic dilatation/aneurysm (32)	0.681 [0.593–0.769]	0.567 [0.505–0.629]	0.567 [0.505–0.629]	0.55 [0.495–0.605]	0.947 [0.926–0.969]
Vertebral compression fractures (51)	0.6 [0.544–0.656]	0.55 [0.508–0.592]	0.56 [0.515–0.605]	0.55 [0.508–0.592]	0.943 [0.913–0.972]
Coronary artery calcification (CAC) (162)	0.838 [0.784–0.893]	0.73 [0.665–0.794]	0.837 [0.788–0.885]	0.864 [0.825–0.903]	0.875 [0.824–0.926]
Lung nodules (96)	0.906 [0.867–0.945]	0.887 [0.845–0.929]	0.849 [0.803–0.895]	0.909 [0.87–0.948]	0.863 [0.818–0.909]
Urolithiasis (22)	0.818 [0.715–0.921]	0.773 [0.666–0.879]	0.795 [0.69–0.901]	0.773 [0.666–0.879]	0.523 [0.478–0.567]
Airspace opacities (infiltrates, consolidations) (144)	0.92 [0.89–0.95]	0.941 [0.915–0.967]	0.948 [0.923–0.973]	0.965 [0.944–0.986]	0.81 [0.757–0.862]
Liver masses (97)	0.969 [0.945–0.993]	0.918 [0.88–0.955]	0.852 [0.806–0.898]	0.928 [0.893–0.963]	0.793 [0.741–0.846]
Renal masses (148)	0.964 [0.94–0.988]	0.949 [0.924–0.974]	0.949 [0.924–0.974]	0.967 [0.945–0.989]	0.778 [0.732–0.824]
Rib fractures (63)	0.574 [0.529–0.619]	0.574 [0.529–0.619]	0.557 [0.517–0.598]	0.541 [0.506–0.576]	0.899 [0.868–0.929]
Pleural effusion (73)	0.942 [0.905–0.979]	0.952 [0.918–0.986]	0.952 [0.918–0.986]	0.938 [0.9–0.976]	0.929 [0.9–0.959]
Pulmonary artery dilatation (54)	0.574 [0.526–0.622]	0.565 [0.52–0.61]	0.528 [0.497–0.559]	0.519 [0.493–0.544]	0.959 [0.929–0.988]
Low vertebral body density (126)	0.504 [0.496–0.513]	0.513 [0.498–0.528]	0.504 [0.496–0.513]	0.509 [0.497–0.521]	0.9 [0.863–0.937]
Adrenal thickening (69)	0.848 [0.793–0.903]	0.75 [0.691–0.81]	0.726 [0.666–0.786]	0.768 [0.709–0.827]	0.849 [0.795–0.902]
Emphysema (98)	0.738 [0.687–0.79]	0.704 [0.654–0.755]	0.742 [0.691–0.793]	0.78 [0.729–0.83]	0.941 [0.914–0.968]
All pathologies (1328)	0.815 [0.802–0.828]	0.790 [0.777–0.804]	0.782 [0.769–0.796]	0.801 [0.788–0.814]	0.883 [0.872–0.894]

**Table 4:** AUC analysis for AI on clinically significant findings only.

Pathology	AUC [95% CI]
Airspace opacities (infiltrates, consolidations)	0.90 [0.85–0.95]
Emphysema	1.00 [0.95–1.00]
Lung nodules	0.97 [0.93–1.00]
Enlarged intrathoracic lymph nodes	0.94 [0.89–0.99]
Pleural effusion	1.00 [0.98–1.00]
Aortic dilatation/aneurysm	1.00 [1.00]
Coronary artery calcification (CAC)	0.96 [0.69–1.00]
Adrenal thickening	0.95 [0.93–1.00]
Rib fractures	0.98 [0.89–1.00]
Vertebral compression fractures	0.73 [0.32–1.00]
Liver masses	0.87 [0.79–0.95]
Renal masses	0.86 [0.69–1.00]
Urolithiasis	0.55 [0.00–1.00]
Average value for all pathologies	0.9 [0.71–1.00]

## Data Availability

Publicly available data from the BIMCV-COVID-19+ initiative were analyzed in this study (https://bimcv.cipf.es/bimcv-projects/bimcv-covid19/—accessed on 16 September 2025). Derived, de-identified result tables and R/GraphPad summaries are available from the corresponding author on reasonable request.

## Supplementary Materials

The following supporting information can be downloaded at: https://www.mdpi.com/article/10.3390/diagnostics15212778/s1, Table S1. Complete raw data from all four radiologists and the AI service across all 229 CT examinations. Contingency table analysis presenting true positives (TP), true negatives (TN), false positives (FP), false negatives (FN), total correct responses (True), and total incorrect responses (False) with percentages and 95% confidence intervals (CI). Data encom-passes all 3,664 individual feature assessments (16 pathological features × 229 examinations). Each row represents the complete diagnostic performance record of each radiologist and the AI service for the entire study dataset, enabling di-rect comparison of detection patterns and error distributions across all readers. Table S2. Detailed raw diagnostic data for each of the 16 individual pathological features, stratified by radiologist and AI service. Contingency table values (TP, TN, FP, FN, True, False) with percentages and 95% CI provided separately for each pathology across all radiologists and AI service. This comprehensive breakdown represents reports at the pathology level, allowing detailed examination of feature-specific accuracy patterns, detection capabilities, and error characteriza-tion for both human readers and the artificial intelligence system across the entire study population.

## Abbreviations

The following abbreviations are used in this manuscript:

AI	Artificial intelligence
CT	Computed tomography
AUC	Area under the ROC curve
ROC	Receiver operating characteristic
CI	Confidence interval
DICOM	Digital Imaging and Communications in Medicine
SR	Structured report
IRB	Institutional review board
APC	Article processing charge
MRMC	Multi-reader multi-case
HU	Hounsfield unit
TP	True positive
FP	False positive
TN	True negative
FN	False negative
CAC	Coronary artery calcification
UMLS	Unified Medical Language System
BIMCV-COVID-19+	Biomedical Imaging and COVID-19 dataset (Valencia region)
