# Supplementary material for: Diagnostic Accuracy of a Multi-Target Artificial Intelligence Service for the Simultaneous Assessment of 16 Pathological Features on Chest and Abdominal CT
Source: Diagnostics (Basel). 2025 Nov 1;15(21):2778. doi: 10.3390/diagnostics15212778 (PMC12607670; doi:10.3390/diagnostics15212778)
Supplement: Supplementary file 1 [file diagnostics-15-02778-s001.zip › Supplementary Table S1.pdf]

	TP	TN	FP	FN	True	False
**All findings**						
Radiologist 1	834<br>22,76%<br>[17,45-28,69]	2337<br>63,78%<br>[57,16-69,99]	23<br>0,63%<br>[0,11-2,41]	470<br>12,83%<br>[8,65-17,68]	3171<br>86,54%<br>[81,34-90,61]	493<br>13,46%<br>[9,39-18,66]
Radiologist 2	772<br>21,07%<br>[15,88-26,81]	2333<br>63,67%<br>[57,16-69,99]	27<br>0,74%<br>[0,11-3,12]	532<br>14,52%<br>[10,13-19,64]	3105<br>84,74%<br>[79,39-89,12]	559<br>15,26%<br>[10,88-20,61]
Radiologist 3	742<br>20,25%<br>[15,1-25,87]	2350<br>64,14%<br>[57,61-70,4]	10<br>0,27%<br>[0,01-2,41]	562<br>15,34%<br>[10,88-20,61]	3092<br>84,39%<br>[78,91-88,74]	572<br>15,61%<br>[11,26-21,09]
Radiologist 4	788<br>21,51%<br>[16,27-27,28]	2355<br>64,27%<br>[57,61-70,4]	5<br>0,14%<br>[0-1,6]	516<br>14,08%<br>[9,76-19,15]	3143<br>85,78%<br>[80,36-89,87]	521<br>14,22%<br>[10,13-19,64]
AI	1143<br>31,2%<br>[25,08-37,43]	2098<br>57,26%<br>[50,52-63,7]	262<br>7,15%<br>[4,05-11,1]	161<br>4,39%<br>[2,11-7,88]	3241<br>88,46%<br>[83,81-92,45]	423<br>11,54%<br>[7,55-16,19]
**Enlarged intrathoracic lymph nodes**						
Radiologist 1	51<br>22,27%<br>[17,05-28,22]	170<br>74,24%<br>[68,06-79,77]	4<br>1,75%<br>[0,48-4,41]	4<br>1,75%<br>[0,48-4,41]	221<br>96,51%<br>[93,23-98,48]	8<br>3,49%<br>[1,52-6,77]
Radiologist 2	52<br>22,71%<br>[17,45-28,69]	171<br>74,67%<br>[68,52-80,17]	3<br>1,31%<br>[0,27-3,78]	3<br>1,31%<br>[0,27-3,78]	223<br>97,38%<br>[94,38-99,03]	6<br>2,62%<br>[0,97-5,62]
Radiologist 3	43<br>18,78%<br>[13,94-24,45]	174<br>75,98%<br>[69,91-81,36]	0<br>0%<br>[0-1,6]	12<br>5,24%<br>[2,74-8,97]	217<br>94,76%<br>[91,03-97,26]	12<br>5,24%<br>[2,74-8,97]
Radiologist 4	48<br>20,96%<br>[15,88-26,81]	174<br>75,98%<br>[69,91-81,36]	0<br>0%<br>[0-1,6]	7<br>3,06%<br>[1,24-6,2]	222<br>96,94%<br>[93,8-98,76]	7<br>3,06%<br>[1,24-6,2]
AI	49<br>21,4%<br>[16,27-27,28]	142<br>62,01%<br>[55,38-68,32]	32<br>13,97%<br>[9,76-19,15]	6<br>2,62%<br>[0,97-5,62]	191<br>83,41%<br>[77,94-87,98]	38<br>16,59%<br>[12,02-22,06]
**Aortic dilatation/aneurysm**						

Radiologist 1	11<br>4,8%<br>[2,42-8,43]	198<br>86,46%<br>[81,34-90,61]	1<br>0,44%<br>[0,01-2,41]	19<br>8,3%<br>[5,07-12,65]	209<br>91,27%<br>[86,83-94,58]	20<br>8,73%<br>[5,42-13,17]
Radiologist 2	4<br>1,75%<br>[0,48-4,41]	199<br>86,9%<br>[81,83-90,98]	0<br>0%<br>[0-1,6]	26<br>11,35%<br>[7,55-16,19]	203<br>88,65%<br>[83,81-92,45]	26<br>11,35%<br>[7,55-16,19]
Radiologist 3	4<br>1,75%<br>[0,48-4,41]	199<br>86,9%<br>[81,83-90,98]	0<br>0%<br>[0-1,6]	26<br>11,35%<br>[7,55-16,19]	203<br>88,65%<br>[83,81-92,45]	26<br>11,35%<br>[7,55-16,19]
Radiologist 4	3<br>1,31%<br>[0,27-3,78]	199<br>86,9%<br>[81,83-90,98]	0<br>0%<br>[0-1,6]	27<br>11,79%<br>[7,92-16,69]	202<br>88,21%<br>[83,31-92,08]	27<br>11,79%<br>[7,92-16,69]
AI	30<br>13,1%<br>[9,02-18,17]	178<br>77,73%<br>[71,78-82,95]	21<br>9,17%<br>[5,77-13,68]	0<br>0%<br>[0-1,6]	208<br>90,83%<br>[86,32-94,23]	21<br>9,17%<br>[5,77-13,68]
**Vertebral compression fractures**						
Radiologist 1	10<br>4,37%<br>[2,11-7,88]	179<br>78,17%<br>[72,25-83,34]	0<br>0%<br>[0-1,6]	40<br>17,47%<br>[12,78-23,02]	189<br>82,53%<br>[76,98-87,22]	40<br>17,47%<br>[12,78-23,02]
Radiologist 2	5<br>2,18%<br>[0,71-5,02]	179<br>78,17%<br>[72,25-83,34]	0<br>0%<br>[0-1,6]	45<br>19,65%<br>[14,71-25,4]	184<br>80,35%<br>[74,6-85,29]	45<br>19,65%<br>[14,71-25,4]
Radiologist 3	6<br>2,62%<br>[0,97-5,62]	179<br>78,17%<br>[72,25-83,34]	0<br>0%<br>[0-1,6]	44<br>19,21%<br>[14,32-24,92]	185<br>80,79%<br>[75,08-85,68]	44<br>19,21%<br>[14,32-24,92]
Radiologist 4	5<br>2,18%<br>[0,71-5,02]	179<br>78,17%<br>[72,25-83,34]	0<br>0%<br>[0-1,6]	45<br>19,65%<br>[14,71-25,4]	184<br>80,35%<br>[74,6-85,29]	45<br>19,65%<br>[14,71-25,4]
AI	49<br>21,4%<br>[16,27-27,28]	162<br>70,74%<br>[64,39-76,55]	17<br>7,42%<br>[4,38-11,62]	1<br>0,44%<br>[0,01-2,41]	211<br>92,14%<br>[87,86-95,28]	18<br>7,86%<br>[4,72-12,14]
**Coronary artery calcification (CAC)**						
Radiologist 1	142<br>62,01%<br>[55,38-68,32]	54<br>23,58%<br>[18,24-29,62]	14<br>6,11%<br>[3,38-10,04]	19<br>8,3%<br>[5,07-12,65]	196<br>85,59%<br>[80,36-89,87]	33<br>14,41%<br>[10,13-19,64]

Radiologist 2	126<br>55,02%<br>[48,33-61,58]	46<br>20,09%<br>[15,1-25,87]	22<br>9,61%<br>[6,12-14,18]	35<br>15,28%<br>[10,88-20,61]	172<br>75,11%<br>[68,99-80,57]	57<br>24,89%<br>[19,43-31,01]
Radiologist 3	125<br>54,59%<br>[47,89-61,16]	61<br>26,64%<br>[21,03-32,86]	7<br>3,06%<br>[1,24-6,2]	36<br>15,72%<br>[11,26-21,09]	186<br>81,22%<br>[75,55-86,06]	43<br>18,78%<br>[13,94-24,45]
Radiologist 4	122<br>53,28%<br>[46,59-59,88]	66<br>28,82%<br>[23,05-35,15]	2<br>0,87%<br>[0,11-3,12]	39<br>17,03%<br>[12,4-22,54]	188<br>82,1%<br>[76,51-86,83]	41<br>17,9%<br>[13,17-23,49]
AI	154<br>67,25%<br>[60,76-73,29]	54<br>23,58%<br>[18,24-29,62]	14<br>6,11%<br>[3,38-10,04]	7<br>3,06%<br>[1,24-6,2]	208<br>90,83%<br>[86,32-94,23]	21<br>9,17%<br>[5,77-13,68]
**Lung nodules**						
Radiologist 1	78<br>34,06%<br>[27,95-40,59]	133<br>58,08%<br>[51,4-64,55]	0<br>0%<br>[0-1,6]	18<br>7,86%<br>[4,72-12,14]	211<br>92,14%<br>[87,86-95,28]	18<br>7,86%<br>[4,72-12,14]
Radiologist 2	75<br>32,75%<br>[26,71-39,24]	132<br>57,64%<br>[50,96-64,12]	1<br>0,44%<br>[0,01-2,41]	21<br>9,17%<br>[5,77-13,68]	207<br>90,39%<br>[85,82-93,88]	22<br>9,61%<br>[6,12-14,18]
Radiologist 3	67<br>29,26%<br>[23,45-35,61]	133<br>58,08%<br>[51,4-64,55]	0<br>0%<br>[0-1,6]	29<br>12,66%<br>[8,65-17,68]	200<br>87,34%<br>[82,32-91,35]	29<br>12,66%<br>[8,65-17,68]
Radiologist 4	80<br>34,93%<br>[28,77-41,49]	131<br>57,21%<br>[50,52-63,7]	2<br>0,87%<br>[0,11-3,12]	16<br>6,99%<br>[4,05-11,1]	211<br>92,14%<br>[87,86-95,28]	18<br>7,86%<br>[4,72-12,14]
AI	82<br>35,81%<br>[29,6-42,39]	116<br>50,66%<br>[43,99-57,3]	17<br>7,42%<br>[4,38-11,62]	14<br>6,11%<br>[3,38-10,04]	198<br>86,46%<br>[81,34-90,61]	31<br>13,54%<br>[9,39-18,66]
**Urolithiasis**						
Radiologist 1	14<br>6,11%<br>[3,38-10,04]	207<br>90,39%<br>[85,82-93,88]	0<br>0%<br>[0-1,6]	8<br>3,49%<br>[1,52-6,77]	221<br>96,51%<br>[93,23-98,48]	8<br>3,49%<br>[1,52-6,77]
Radiologist 2	12<br>5,24%<br>[2,74-8,97]	207<br>90,39%<br>[85,82-93,88]	0<br>0%<br>[0-1,6]	10<br>4,37%<br>[2,11-7,88]	219<br>95,63%<br>[92,12-97,89]	10<br>4,37%<br>[2,11-7,88]
Radiologist 3	13<br>5,68%<br>[3,06-9,51]	207<br>90,39%<br>[85,82-93,88]	0<br>0%<br>[0-1,6]	9<br>3,93%<br>[1,81-7,33]	220<br>96,07%<br>[92,67-98,19]	9<br>3,93%<br>[1,81-7,33]
Radiologist 4	12<br>5,24%<br>[2,74-8,97]	207<br>90,39%<br>[85,82-93,88]	0<br>0%<br>[0-1,6]	10<br>4,37%<br>[2,11-7,88]	219<br>95,63%<br>[92,12-97,89]	10<br>4,37%<br>[2,11-7,88]

AI	1<br>0,44%<br>[0,01-2,41]	207<br>90,39%<br>[85,82-93,88]	0<br>0%<br>[0-1,6]	21<br>9,17%<br>[5,77-13,68]	208<br>90,83%<br>[86,32-94,23]	21<br>9,17%<br>[5,77-13,68]
**Airspace opacities (infiltrates, consolidations)**						
Radiologist 1	121<br>52,84%<br>[46,15-59,45]	85<br>37,12%<br>[30,85-43,73]	0<br>0%<br>[0-1,6]	23<br>10,04%<br>[6,47-14,69]	206<br>89,96%<br>[85,31-93,53]	23<br>10,04%<br>[6,47-14,69]
Radiologist 2	127<br>55,46%<br>[48,77-62,01]	85<br>37,12%<br>[30,85-43,73]	0<br>0%<br>[0-1,6]	17<br>7,42%<br>[4,38-11,62]	212<br>92,58%<br>[88,38-95,62]	17<br>7,42%<br>[4,38-11,62]
Radiologist 3	129<br>56,33%<br>[49,64-62,85]	85<br>37,12%<br>[30,85-43,73]	0<br>0%<br>[0-1,6]	15<br>6,55%<br>[3,71-10,57]	214<br>93,45%<br>[89,43-96,29]	15<br>6,55%<br>[3,71-10,57]
Radiologist 4	134<br>58,52%<br>[51,84-64,97]	85<br>37,12%<br>[30,85-43,73]	0<br>0%<br>[0-1,6]	10<br>4,37%<br>[2,11-7,88]	219<br>95,63%<br>[92,12-97,89]	10<br>4,37%<br>[2,11-7,88]
AI	140<br>61,14%<br>[54,49-67,49]	55<br>24,02%<br>[18,64-30,09]	30<br>13,1%<br>[9,02-18,17]	4<br>1,75%<br>[0,48-4,41]	195<br>85,15%<br>[79,88-89,49]	34<br>14,85%<br>[10,51-20,12]
**Liver masses**						
Radiologist 1	91<br>39,74%<br>[33,35-46,39]	132<br>57,64%<br>[50,96-64,12]	0<br>0%<br>[0-1,6]	6<br>2,62%<br>[0,97-5,62]	223<br>97,38%<br>[94,38-99,03]	6<br>2,62%<br>[0,97-5,62]
Radiologist 2	81<br>35,37%<br>[29,19-41,94]	132<br>57,64%<br>[50,96-64,12]	0<br>0%<br>[0-1,6]	16<br>6,99%<br>[4,05-11,1]	213<br>93,01%<br>[88,9-95,95]	16<br>6,99%<br>[4,05-11,1]
Radiologist 3	69<br>30,13%<br>[24,26-36,52]	131<br>57,21%<br>[50,52-63,7]	1<br>0,44%<br>[0,01-2,41]	28<br>12,23%<br>[8,28-17,18]	200<br>87,34%<br>[82,32-91,35]	29<br>12,66%<br>[8,65-17,68]
Radiologist 4	83<br>36,24%<br>[30,01-42,84]	132<br>57,64%<br>[50,96-64,12]	0<br>0%<br>[0-1,6]	14<br>6,11%<br>[3,38-10,04]	215<br>93,89%<br>[89,96-96,62]	14<br>6,11%<br>[3,38-10,04]
AI	65<br>28,38%<br>[22,64-34,7]	121<br>52,84%<br>[46,15-59,45]	11<br>4,8%<br>[2,42-8,43]	32<br>13,97%<br>[9,76-19,15]	186<br>81,22%<br>[75,55-86,06]	43<br>18,78%<br>[13,94-24,45]
**Renal masses**						

Radiologist 1	140<br>61,14%<br>[54,49-67,49]	80<br>34,93%<br>[28,77-41,49]	2<br>0,87%<br>[0,11-3,12]	7<br>3,06%<br>[1,24-6,2]	220<br>96,07%<br>[92,67-98,19]	9<br>3,93%<br>[1,81-7,33]
Radiologist 2	132<br>57,64%<br>[50,96-64,12]	82<br>35,81%<br>[29,6-42,39]	0<br>0%<br>[0-1,6]	15<br>6,55%<br>[3,71-10,57]	214<br>93,45%<br>[89,43-96,29]	15<br>6,55%<br>[3,71-10,57]
Radiologist 3	132<br>57,64%<br>[50,96-64,12]	82<br>35,81%<br>[29,6-42,39]	0<br>0%<br>[0-1,6]	15<br>6,55%<br>[3,71-10,57]	214<br>93,45%<br>[89,43-96,29]	15<br>6,55%<br>[3,71-10,57]
Radiologist 4	139<br>60,7%<br>[54,05-67,07]	81<br>35,37%<br>[29,19-41,94]	1<br>0,44%<br>[0,01-2,41]	8<br>3,49%<br>[1,52-6,77]	220<br>96,07%<br>[92,67-98,19]	9<br>3,93%<br>[1,81-7,33]
AI	89<br>38,86%<br>[32,51-45,51]	78<br>34,06%<br>[27,95-40,59]	4<br>1,75%<br>[0,48-4,41]	58<br>25,33%<br>[19,83-31,48]	167<br>72,93%<br>[66,68-78,57]	62<br>27,07%<br>[21,43-33,32]
**Rib fractures**						
Radiologist 1	9<br>3,93%<br>[1,81-7,33]	168<br>73,36%<br>[67,14-78,97]	0<br>0%<br>[0-1,6]	52<br>22,71%<br>[17,45-28,69]	177<br>77,29%<br>[71,31-82,55]	52<br>22,71%<br>[17,45-28,69]
Radiologist 2	9<br>3,93%<br>[1,81-7,33]	168<br>73,36%<br>[67,14-78,97]	0<br>0%<br>[0-1,6]	52<br>22,71%<br>[17,45-28,69]	177<br>77,29%<br>[71,31-82,55]	52<br>22,71%<br>[17,45-28,69]
Radiologist 3	7<br>3,06%<br>[1,24-6,2]	168<br>73,36%<br>[67,14-78,97]	0<br>0%<br>[0-1,6]	54<br>23,58%<br>[18,24-29,62]	175<br>76,42%<br>[70,38-81,76]	54<br>23,58%<br>[18,24-29,62]
Radiologist 4	5<br>2,18%<br>[0,71-5,02]	168<br>73,36%<br>[67,14-78,97]	0<br>0%<br>[0-1,6]	56<br>24,45%<br>[19,03-30,55]	173<br>75,55%<br>[69,45-80,97]	56<br>24,45%<br>[19,03-30,55]
AI	61<br>26,64%<br>[21,03-32,86]	134<br>58,52%<br>[51,84-64,97]	34<br>14,85%<br>[10,51-20,12]	0<br>0%<br>[0-1,6]	195<br>85,15%<br>[79,88-89,49]	34<br>14,85%<br>[10,51-20,12]
**Pleural effusion**						
Radiologist 1	65<br>28,38%<br>[22,64-34,7]	155<br>67,69%<br>[61,21-73,7]	1<br>0,44%<br>[0,01-2,41]	8<br>3,49%<br>[1,52-6,77]	220<br>96,07%<br>[92,67-98,19]	9<br>3,93%<br>[1,81-7,33]
Radiologist 2	66<br>28,82%<br>[23,05-35,15]	156<br>68,12%<br>[61,66-74,11]	0<br>0%<br>[0-1,6]	7<br>3,06%<br>[1,24-6,2]	222<br>96,94%<br>[93,8-98,76]	7<br>3,06%<br>[1,24-6,2]

Radiologist 3	66<br>28,82%<br>[23,05-35,15]	156<br>68,12%<br>[61,66-74,11]	0<br>0%<br>[0-1,6]	7<br>3,06%<br>[1,24-6,2]	222<br>96,94%<br>[93,8-98,76]	7<br>3,06%<br>[1,24-6,2]
Radiologist 4	64<br>27,95%<br>[22,24-34,24]	156<br>68,12%<br>[61,66-74,11]	0<br>0%<br>[0-1,6]	9<br>3,93%<br>[1,81-7,33]	220<br>96,07%<br>[92,67-98,19]	9<br>3,93%<br>[1,81-7,33]
AI	72<br>31,44%<br>[25,49-37,89]	136<br>59,39%<br>[52,72-65,81]	20<br>8,73%<br>[5,42-13,17]	1<br>0,44%<br>[0,01-2,41]	208<br>90,83%<br>[86,32-94,23]	21<br>9,17%<br>[5,77-13,68]
**Pulmonary artery dilatation**						
Radiologist 1	8<br>3,49%<br>[1,52-6,77]	175<br>76,42%<br>[70,38-81,76]	0<br>0%<br>[0-1,6]	46<br>20,09%<br>[15,1-25,87]	183<br>79,91%<br>[74,13-84,9]	46<br>20,09%<br>[15,1-25,87]
Radiologist 2	7<br>3,06%<br>[1,24-6,2]	175<br>76,42%<br>[70,38-81,76]	0<br>0%<br>[0-1,6]	47<br>20,52%<br>[15,49-26,34]	182<br>79,48%<br>[73,66-84,51]	47<br>20,52%<br>[15,49-26,34]
Radiologist 3	3<br>1,31%<br>[0,27-3,78]	175<br>76,42%<br>[70,38-81,76]	0<br>0%<br>[0-1,6]	51<br>22,27%<br>[17,05-28,22]	178<br>77,73%<br>[71,78-82,95]	51<br>22,27%<br>[17,05-28,22]
Radiologist 4	2<br>0,87%<br>[0,11-3,12]	175<br>76,42%<br>[70,38-81,76]	0<br>0%<br>[0-1,6]	52<br>22,71%<br>[17,45-28,69]	177<br>77,29%<br>[71,31-82,55]	52<br>22,71%<br>[17,45-28,69]
AI	52<br>22,71%<br>[17,45-28,69]	167<br>72,93%<br>[66,68-78,57]	8<br>3,49%<br>[1,52-6,77]	2<br>0,87%<br>[0,11-3,12]	219<br>95,63%<br>[92,12-97,89]	10<br>4,37%<br>[2,11-7,88]
**Low vertebral body density**						
Radiologist 1	1<br>0,44%<br>[0,01-2,41]	115<br>50,22%<br>[43,56-56,87]	0<br>0%<br>[0-1,6]	113<br>49,34%<br>[42,7-56,01]	116<br>50,66%<br>[43,99-57,3]	113<br>49,34%<br>[42,7-56,01]
Radiologist 2	3<br>1,31%<br>[0,27-3,78]	115<br>50,22%<br>[43,56-56,87]	0<br>0%<br>[0-1,6]	111<br>48,47%<br>[41,84-55,15]	118<br>51,53%<br>[44,85-58,16]	111<br>48,47%<br>[41,84-55,15]
Radiologist 3	1<br>0,44%<br>[0,01-2,41]	115<br>50,22%<br>[43,56-56,87]	0<br>0%<br>[0-1,6]	113<br>49,34%<br>[42,7-56,01]	116<br>50,66%<br>[43,99-57,3]	113<br>49,34%<br>[42,7-56,01]
Radiologist 4	2<br>0,87%<br>[0,11-3,12]	115<br>50,22%<br>[43,56-56,87]	0<br>0%<br>[0-1,6]	112<br>48,91%<br>[42,27-55,58]	117<br>51,09%<br>[44,42-57,73]	112<br>48,91%<br>[42,27-55,58]

AI	113<br>49,34%<br>[42,7-56,01]	93<br>40,61%<br>[34,19-47,28]	22<br>9,61%<br>[6,12-14,18]	1<br>0,44%<br>[0,01-2,41]	206<br>89,96%<br>[85,31-93,53]	23<br>10,04%<br>[6,47-14,69]
**Adrenal thickening**						
Radiologist 1	48<br>20,96%<br>[15,88-26,81]	160<br>69,87%<br>[63,48-75,74]	0<br>0%<br>[0-1,6]	21<br>9,17%<br>[5,77-13,68]	208<br>90,83%<br>[86,32-94,23]	21<br>9,17%<br>[5,77-13,68]
Radiologist 2	35<br>15,28%<br>[10,88-20,61]	159<br>69,43%<br>[63,02-75,33]	1<br>0,44%<br>[0,01-2,41]	34<br>14,85%<br>[10,51-20,12]	194<br>84,72%<br>[79,39-89,12]	35<br>15,28%<br>[10,88-20,61]
Radiologist 3	32<br>13,97%<br>[9,76-19,15]	158<br>69%<br>[62,57-74,92]	2<br>0,87%<br>[0,11-3,12]	37<br>16,16%<br>[11,64-21,58]	190<br>82,97%<br>[77,46-87,6]	39<br>17,03%<br>[12,4-22,54]
Radiologist 4	37<br>16,16%<br>[11,64-21,58]	160<br>69,87%<br>[63,48-75,74]	0<br>0%<br>[0-1,6]	32<br>13,97%<br>[9,76-19,15]	197<br>86,03%<br>[80,85-90,24]	32<br>13,97%<br>[9,76-19,15]
AI	55<br>24,02%<br>[18,64-30,09]	144<br>62,88%<br>[56,27-69,15]	16<br>6,99%<br>[4,05-11,1]	14<br>6,11%<br>[3,38-10,04]	199<br>86,9%<br>[81,83-90,98]	30<br>13,1%<br>[9,02-18,17]
**Emphysema**						
Radiologist 1	45<br>19,65%<br>[14,71-25,4]	135<br>58,95%<br>[52,28-65,39]	1<br>0,44%<br>[0,01-2,41]	48<br>20,96%<br>[15,88-26,81]	180<br>78,6%<br>[72,72-83,73]	49<br>21,4%<br>[16,27-27,28]
Radiologist 2	38<br>16,59%<br>[12,02-22,06]	136<br>59,39%<br>[52,72-65,81]	0<br>0%<br>[0-1,6]	55<br>24,02%<br>[18,64-30,09]	174<br>75,98%<br>[69,91-81,36]	55<br>24,02%<br>[18,64-30,09]
Radiologist 3	45<br>19,65%<br>[14,71-25,4]	136<br>59,39%<br>[52,72-65,81]	0<br>0%<br>[0-1,6]	48<br>20,96%<br>[15,88-26,81]	181<br>79,04%<br>[73,19-84,12]	48<br>20,96%<br>[15,88-26,81]
Radiologist 4	52<br>22,71%<br>[17,45-28,69]	136<br>59,39%<br>[52,72-65,81]	0<br>0%<br>[0-1,6]	41<br>17,9%<br>[13,17-23,49]	188<br>82,1%<br>[76,51-86,83]	41<br>17,9%<br>[13,17-23,49]
AI	93<br>40,61%<br>[34,19-47,28]	120<br>52,4%<br>[45,72-59,02]	16<br>6,99%<br>[4,05-11,1]	0<br>0%<br>[0-1,6]	213<br>93,01%<br>[88,9-95,95]	16<br>6,99%<br>[4,05-11,1]
**Epicardial fat (increased)**						
Radiologist 1	0<br>0%<br>[0-1,6]	191<br>83,41%<br>[77,94-87,98]	0<br>0%<br>[0-1,6]	38<br>16,59%<br>[12,02-22,06]	191<br>83,41%<br>[77,94-87,98]	38<br>16,59%<br>[12,02-22,06]

Radiologist 2	0<br>0%<br>[0-1,6]	191<br>83,41%<br>[77,94-87,98]	0<br>0%<br>[0-1,6]	38<br>16,59%<br>[12,02-22,06]	191<br>83,41%<br>[77,94-87,98]	38<br>16,59%<br>[12,02-22,06]
Radiologist 3	0<br>0%<br>[0-1,6]	191<br>83,41%<br>[77,94-87,98]	0<br>0%<br>[0-1,6]	38<br>16,59%<br>[12,02-22,06]	191<br>83,41%<br>[77,94-87,98]	38<br>16,59%<br>[12,02-22,06]
Radiologist 4	0<br>0%<br>[0-1,6]	191<br>83,41%<br>[77,94-87,98]	0<br>0%<br>[0-1,6]	38<br>16,59%<br>[12,02-22,06]	191<br>83,41%<br>[77,94-87,98]	38<br>16,59%<br>[12,02-22,06]
AI	38<br>16,59%<br>[12,02-22,06]	191<br>83,41%<br>[77,94-87,98]	0<br>0%<br>[0-1,6]	0<br>0%<br>[0-1,6]	229<br>100%<br>[98,4-100]	0<br>0%<br>[0-1,6]
